# Prospective Monitoring of Serum Values of CBC, Total IgE, Thyroid Findings, D-Dimer, Vitamin D, and Inflammatory Molecules CRP, ESR, and IL-6 and Clinical Features of Chronic Spontaneous Urticaria Patients During Antihistamine Treatment

**DOI:** 10.3390/ijms27052503

**Published:** 2026-03-09

**Authors:** Matea Kuna, Mario Štefanović, Ema Barac, Fran Ivan Madunić, Milena Hanžek, Liborija Lugović-Mihić

**Affiliations:** 1 Department of Dermatovenereology, University Hospital Center “Sestre Milosrdnice”, Vinogradska 29, 10000 Zagreb, Croatia; matea.kuna43@gmail.com (M.K.); ema.barac@gmail.com (E.B.); 2 School of Dental Medicine, University of Zagreb, 10000 Zagreb, Croatia; 3 Department of Clinical Chemistry, University Hospital Center “Sestre Milosrdnice”, Vinogradska 29, 10000 Zagreb, Croatia; milenanjegovan13@gmail.com; 4 Department of Oncology and Nuclear Medicine, University Hospital Center “Sestre Milosrdnice”, Vinogradska 29, 10000 Zagreb, Croatia; fimadunic@gmail.com

**Keywords:** biomarkers, chronic spontaneous urticaria, diagnostics, quality of life, blood cell count (CBC), thyroid findings, vitamin D, D-dimer, IL-6, ESR, CRP

## Abstract

Having appropriate and meaningful diagnostic procedures is crucial in the approach to patients with chronic spontaneous urticaria (CSU), so we wanted to investigate relationships between CSU patients’ common serum factors and clinical CSU features, and their temporal trends during antihistamine treatment. In this exploratory hypothesis-based study, we assessed disease severity and quality of life (QoL) in, initially, 41 CSU patients using UAS7, daily UAS, UCT, DLQI, and CU-Q2oL. Concurrently, we measured serum complete blood count (CBC), total IgE, thyroid antibodies and hormones, ANA, D-dimer, vitamin D, and the inflammatory molecules CRP, ESR and IL-6. We compared initial (T1) and follow-up findings (T2) (after 3 months of antihistamine therapy). Basophil concentration was the only examined serum factor useful in assessing current CSU severity/daily UAS (sensitivity 78.6%; specificity 63%; *p* = 0.028). Basopenia was more frequent in patients with moderate/severe CSU than in those with mild disease or remission, as measured by daily UAS (79% vs. 37%; *p* = 0.020). T4 values showed a significant dependence on CSU duration (r = −0.328; *p* = 0.036). ESR was the only examined serum factor significantly associated with weekly CSU severity (UAS7) (*p* = 0.038). Antihistamine treatment significantly reduced CSU activity (recorded by daily UAS and UAS7) and improved QoL (DLQI) (*p* = 0.006) and disease control/UCT (*p* = 0.005). After three months of treatment, only the CRP value correlated with CSU control/UCT (*p* = 0.014). We encourage the use of diagnostics employing basophil counts and clinical indices UAS7, daily UAS, UCT and DLQI for insight into a patient’s CSU clinical condition. Serum factor values did not change during the 3-month treatment period, so it is not useful to measure them repeatedly. Although this study involved a small cohort and has many limitations, these promising results highlight the need for replication with a greater number of CSU patients.

## 1. Introduction

Chronic urticaria (CU) is an inflammatory skin disease marked by recurrent wheals and pruritus, with or without angioedema, for more than six weeks [1]. CU is classified into chronic spontaneous urticaria (CSU), with no identifiable cause, and chronic inducible urticaria (CIU) [2]. Its lifetime prevalence is about 4.4% [3]. The yearly incidence is approximately 1.4% [4]. CSU in adults most commonly begins between 30 and 50 years of age and is more frequent in women (pediatric CSU is less common; 0.1–0.3%) [5,6]. In most cases, CU is a self-limiting disorder, but in approximately 20% of patients, the condition may persist beyond five years [7]. In the majority of cases, the underlying cause of CSU remains unknown. Clinical symptoms are unpredictable and sudden, with wheals typically recurring and treatment resistance frequently observed [8].

Adequate management and appropriate diagnostic procedures are crucial in the approach to patients with CSU. Routine laboratory investigations should include a complete blood count (CBC) and/or C-reactive protein (CRP), as well as thyroid findings and total serum IgE in CSU patients, with additional tests being optional [1]. Several serum molecules/factors have been identified as indicators of CSU activity, such as CRP, erythrocyte sedimentation rate (ESR), and the cytokine IL-6 [9,10,11,12]. Among CBC values, basopenia is an especially valuable disease-related indicator associated with high CSU severity. Also, serum inflammatory molecules CRP and IL-6 have been shown to strongly correlate with CSU severity [13]. Previous studies have yielded a few observations: CSU patients resistant to second-generation antihistamines had elevated CRP values [14]; patients with lower CRP values had better responses to treatment [15]; and during exacerbation and remission there was no significant difference in CRP values [16]. Patients with CSU also demonstrate systemic inflammation, with increased CRP, IL-6, IL-10, and tumor necrosis factor-alpha (TNF-α), as well as higher cortisol values and a lower perception of stress than in healthy controls [17]. Moreover, an imbalance was observed between pro-inflammatory and anti-inflammatory adipokines secreted by adipocytes, which may act as contributing factors in the development or exacerbation of CSU and angioedema [18].

Studies investigating CSU severity in relation to serum factors have demonstrated an association with basophil counts and clinical manifestations of CU. Peripheral blood basopenia correlates with urticaria symptom intensity, while skin basophil infiltration contributes to the severity of cutaneous symptoms [19,20,21]. Basophil reactivity may be presented with their FcεRI receptor density, which was supported by two studies where patients treated with omalizumab had a significant reduction in FcεRI receptor density on basophils [22,23]. In addition, one study showed significantly higher D-dimer and fibrinogen levels in patients with urticaria associated with angioedema and significant decreases in D-dimer values after the first administration of omalizumab [24,25]. Numerous studies support low total IgE levels as a predictor of nonresponse or poor response to omalizumab, where low baseline IgE values were related with nonresponse to omalizumab [26,27,28].

According to the literature, there are several promising factors (potential predictors) related to nonresponse of CSU patients to antihistamines (high UAS7, D-dimer, elevated CRP, concomitant CIU, previous corticosteroid treatment, and low CU-Q2oL scores), predictors of nonresponse to omalizumab (low total IgE levels), and predictors of response to cyclosporine (positive basophil histamine release assay results and low total IgE levels) [29].

Whereas in our previous pilot study with CSU patients we analyzed three serum inflammatory molecules (CRP, ESR, IL-6) in comparison with clinical CSU findings, in this exploratory prospective study (primarily exploratory and hypothesis-generating research) we aimed to examine other serum factors (CBC, total IgE, anti-TPO, anti-TG, TSH, T3, T4, ANA, D-dimer and vitamin D) and their relationship with clinical CSU features measured by verified indices [30]. We aimed to examine whether the values of total IgE, ANA, thyroid findings and vitamin D change significantly during a three-month prospective follow-up and antihistamine treatment of CSU patients. We also examined longitudinal trends/variations in examined serum factors and clinical CSU indices during CSU treatment, as well as their relationship. In addition, we looked at the temporal characteristics of the investigated factors, i.e., which serum factors are associated with a patient’s current state/daily UAS and which with the weekly disease state/weekly UAS.

## 2. Results

### 2.1. Study Population

**Figure 1:**
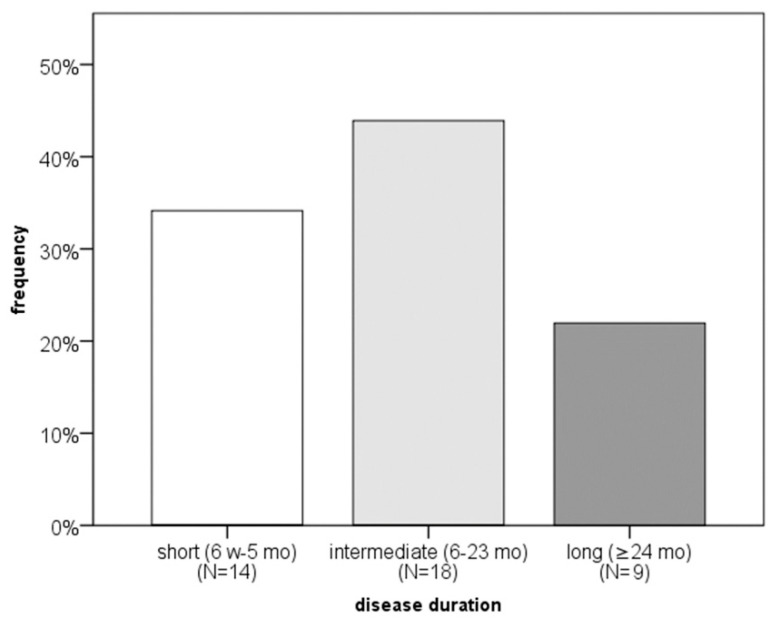
Distribution of CSU patients (percentage) according to disease duration. N—number of patients with CSU, w—weeks, mo—months.

The study included 41 CSU patients (32 women, nine men), aged 23–79 years, 44% of whom had disease lasting 3–6 months (Figure 1). All examinees were ≥18, free of autoimmune or chronic diseases, and not on immunomodulators, psychiatric drugs, or biologics, and all subjects received bilastine (20 mg twice daily, up to 4 tablets/day if needed). Only three of 41 had prior systemic corticosteroid use, discontinued 2–4 weeks before enrollment.

Initially, for statistical analysis purposes for our longitudinal study, we calculated that we needed a minimum sample size of 28 participants. We assumed there would be an expected treatment-induced difference of five participants for the non-standard IL-6 molecule, a standard deviation of nine, a statistical power of 80%, and a significance level of 0.05. To account for an anticipated 20% dropout rate, the sample size was increased accordingly. The calculation was performed using the online calculator available at https://statulator.com/SampleSize/ss2PM.html, accessed on 15 October 2021.

The initial sample, at the first measurement (T1 point), consisted of 41 patients with CSU. In the prospective follow-up, 32% of patients dropped out of the study after 3 months (T2 point); some were unable to come to the scheduled appointment within 3 months or subsequently met exclusion criteria such as the use of systemic corticosteroids. Thus, at the second measurement, there were a total of 28 patients with CSU. The majority had all examined serum factors.

**Figure 2:**
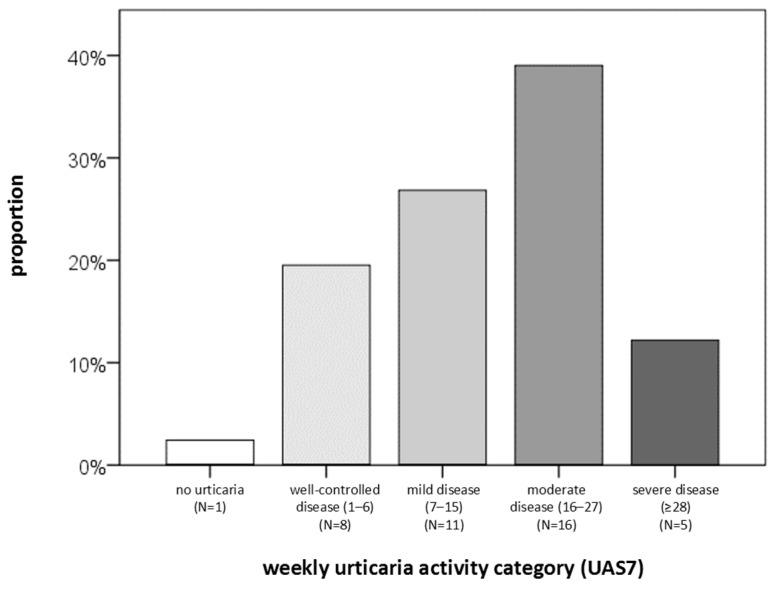
Distribution of CSU patients (percentage) according to weekly disease activity (UAS7). N—number of patients with CSU.

**Figure 3:**
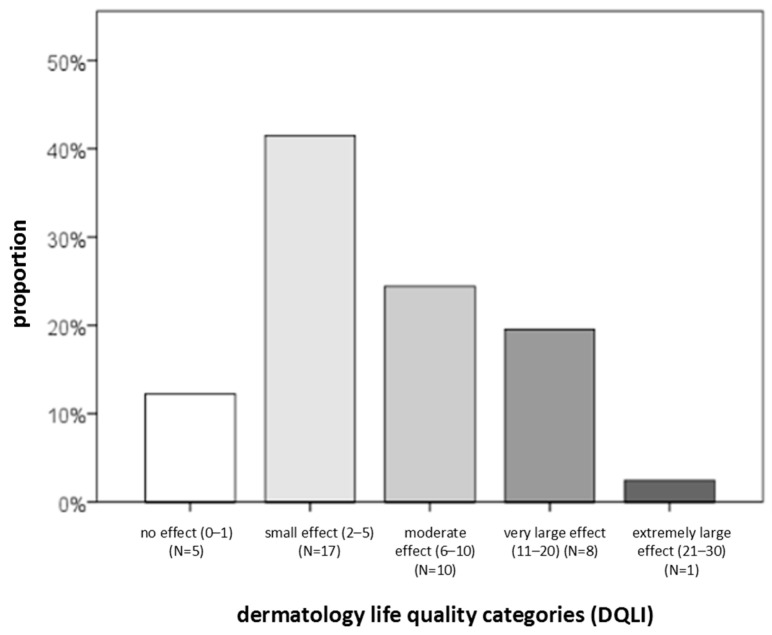
Distribution of CSU patients (percentage) according to different levels of impact of the disease on DLQI. N—number of patients with CSU.

**Figure 4:**
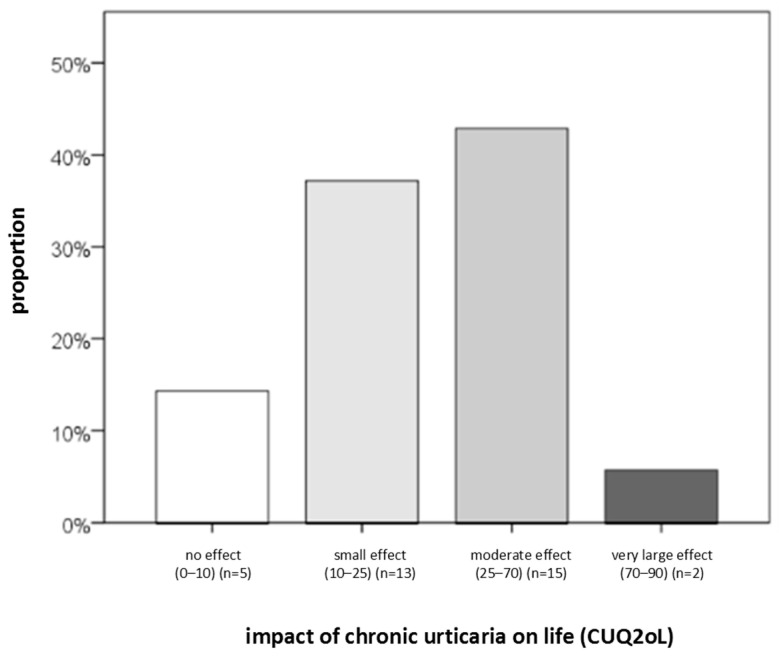
Distribution of CSU patients (percentage) according to CSU-specific QoL impact (CU-Q2oL). N—number of patients with CSU.

Regarding CSU severity, QoL, and disease control, the following findings were obtained. The largest proportion of patients had moderate disease (39%) and reported a small impact of the disease on DLQI (42%) (Figure 2, Figure 3 and Figure 4). Severe disease was present in only five patients, while one patient was in remission without urticaria (according to UAS7 severity groups). At the same time, 90% of participants had uncontrolled disease, and 43% reported a moderate impact of urticaria on their QoL (Figure 4). Based on DLQI scoring, only one patient experienced an extremely large impact of CSU on DLQI. According to CU-Q2oL categories, two patients reported a very large CSU-specific QoL impairment, and none of the patients achieved complete disease control (UCT). Due to technical limitations and demands of individual patients, it was not possible to obtain a full dataset of all examined serum factors for every individual. (The dropout rate after three months was 32%; i.e., 28 out of 41 subjects came to the second follow-up, point T2.) Some serum factors were missing in a few cases due to technical reasons occurring predominantly during laboratory processes.

### 2.2. Descriptive Statistics

The sample characteristics are presented in Table 1.

### 2.3. Relationship Between Examined Serum Factors, Age, Gender, CSU Duration, Severity, Control, and QoL (At T1 Time Point and T2 Control Time Point)

Male and female patients did not significantly differ in age. The only difference between sexes was in UCT, which was higher in females than males (*p* = 0.041). Age was not associated with disease duration or severity. When age and sex were together analyzed, neither influenced disease duration or severity.

When looking at serum parameter levels, basophil counts were decreased in 21/41 (51%), while others were mostly within the following ranges: TSH in 35/40 (88%), T3 in 39/41 (95%), T4 in 37/41 (90%), CRP in 33/41 (81%), ESR in 29/41 (71%), IL-6 in 36/40 (90%), D-dimer in 29/39 (74%), and vitamin D enough or optimal in 29/41 (71%). Age was related to vitamin D and ESR (Table 2). Regarding serum parameter levels, only T4 was related to disease duration (r = −0.328; *p* = 0.036; Table 2). According to the Kruskall–Wallis test, serum parameter levels did not differ between the categories of disease duration (*p* = 0.177–0.918). In females, UCT was higher (*p* = 0.041), while T3 was lower than in males (*p* = 0.016).

When examining the relationship between the CSU severity indicators, i.e., the relationship between daily UAS and weekly UAS (UAS7), and the studied factors, daily UAS was associated with basophil values (r = 0.457; *p* = 0.002) but not with D-dimer, CRP or IL-6.

Regarding the weekly UAS7 in relation to the other studied factors, UAS7 was associated with UCT (r = −0.673; *p* < 0.001), CU-Q2oL (r = 0.608; *p* < 0.001) and DLQI (r = 0.776; *p* < 0.001), but was not associated with ESR.

DLQI correlated better with UAS7 than CUQ2oL (r = 0.778 vs. 0.608) and UCT (r = −0.669 vs. −0.558), and less with daily UAS (r = 0.439 vs. 0.518), although no statistically significant difference was demonstrated between DLQI and CUQ2oL.

None of the factors examined correlated with CSU duration.

#### 2.3.1. Results for CBC (Basophils) Measured at T1 and at the Three-Month Follow-Up (T2)

**Figure 5:**
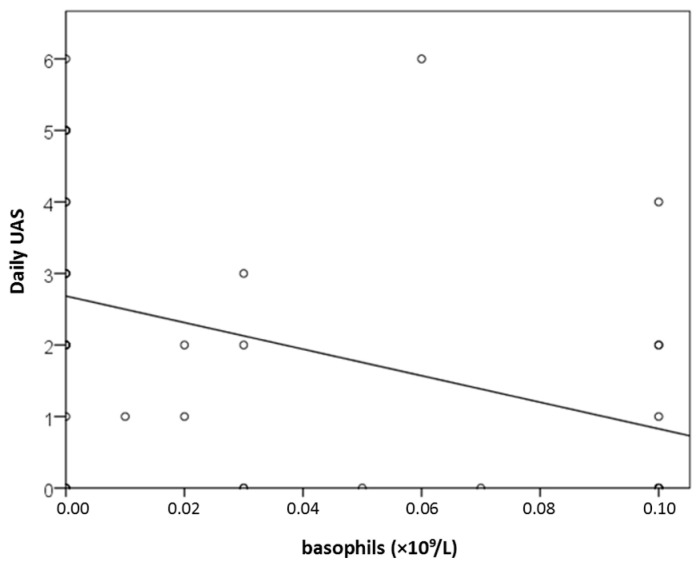
Association between basophil count (×10^9^/L) and daily UAS (*p* = 0.002).

**Figure 6:**
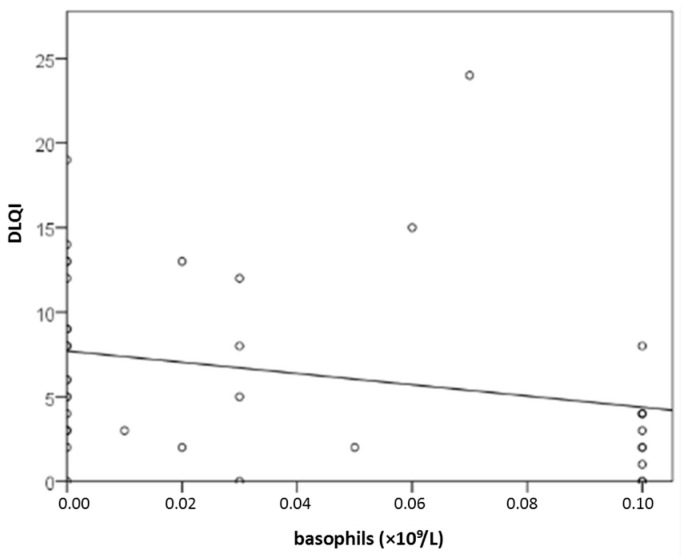
Association between basophil count (×10^9^/L) and DLQI impairment (*p* ≤ 0.034).

At the initial measurement (T1), basophil concentration was significantly associated with daily disease activity (daily UAS) (r = −0.475; *p* = 0.002), revealing a negative linear correlation, although no significant association was observed with UAS7 (Figure 5). With each increase in basophils by 1 × 10^9^/L, daily UAS decreased by 18.6 points (y = 2.7 − 18.6x; Figure 5). Basophil counts were also significantly inversely correlated with DLQI (r = −0.358 to −0.359; *p* ≤ 0.034; Figure 6), but not with disease control (UCT).

**Figure 7:**
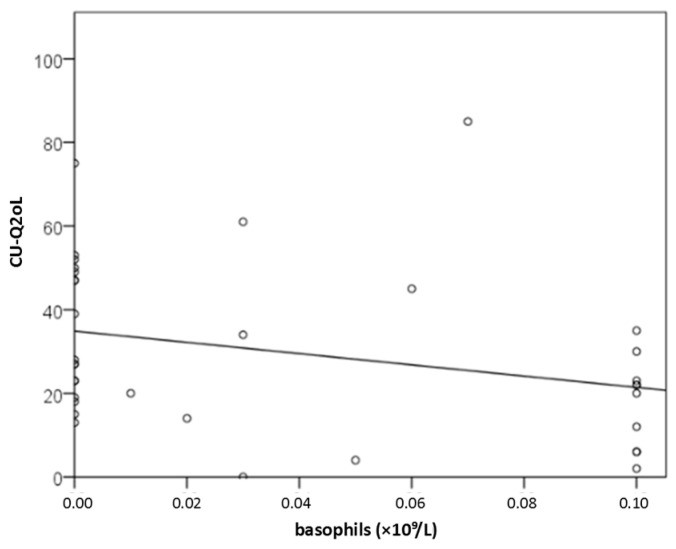
Association between basophil count (×10^9^/L) and CU-Q2oL impairment (*p* ≤ 0.023).

Basophils may serve as serum factors associated with dermatology-specific QoL impairment (DLQI): with each increase of 1 × 10^9^/L, DLQI decreased by 33.3 points (y = 7.7 − 33.3x; Figure 6). The association between basophil concentration and CU-specific QoL impairment (CU-Q2oL) was stronger than that between the proportion of basophils in blood and CU-specific QoL impairment (r = −0.385 and r = −0.359; *p* ≤ 0.023; Figure 7). With increasing CSU activity and CU-Q2oL, basophil levels decreased. Basopenia was more common in CSU patients with moderate or severe CSU than in patients with mild CSU or remission, as measured by daily UAS (79% vs. 37%; *p* = 0.020). However, no significant differences were found when comparing dichotomized groups using other questionnaires. In linear regression when controlling for the effect of age and gender, basophils lost their significance and were not related to DLQI or CU-Q2oL. However, they retained a significant relationship with daily UAS when age and gender were controlled for (*p* = 0.008).

**Figure 8:**
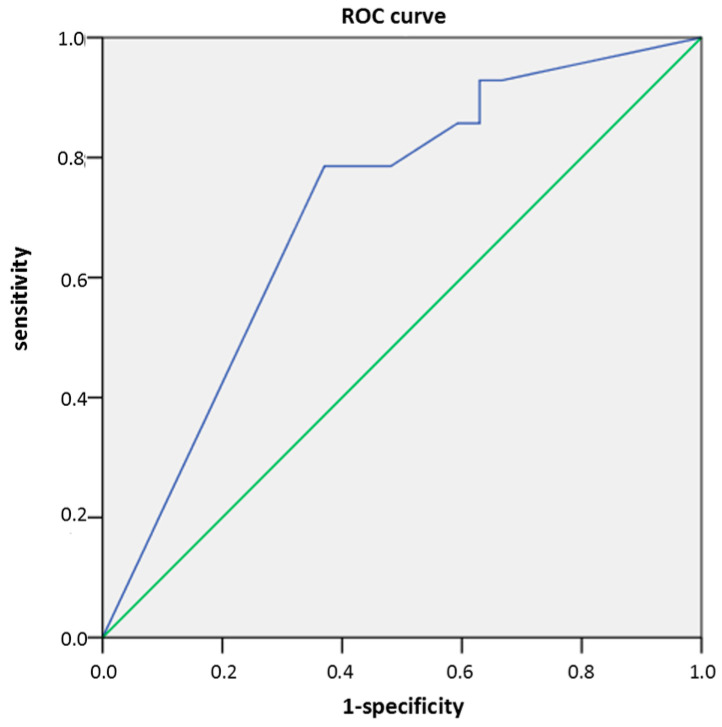
Sensitivity and specificity curve of basophil count in CSU patients for assessing CSU clinical severity measured by daily UAS (*p* = 0.028). *p* = level of statistical significance.

According to logistic regression, basopenia was associated with a 6.2-fold increased likelihood of moderate or severe CSU (OR 6.2; 95% CI 1.4–27.8; *p* = 0.017). Basophil concentration was the only examined serum factor associated with current CSU severity measured by daily UAS, with a sensitivity of 78.6%, specificity of 63%, and AUC of 0.71 (*p* = 0.028; Figure 8). Basophil concentration demonstrated moderate accuracy, being more effective in detecting severe CSU but less effective in identifying milder forms of the disease.

After three months of antihistamine therapy, basophils did not change, as measured at T2 (Table 3).

#### 2.3.2. Results for Total IgE

In this prospective study, total serum IgE was measured in all participants, with a mean value of 60.3 kIU/L (range: 23.0–113.0 kIU/L). Serum D-dimer levels showed no significant correlation with disease duration, severity, or control of CSU, nor with patients’ QoL or other examined serum factors associated with CSU.

After three months of standard antihistamine therapy, at T2, values of total IgE did not differ significantly from baseline values (Table 3).

#### 2.3.3. Results for Thyroid Findings

**Figure 9:**
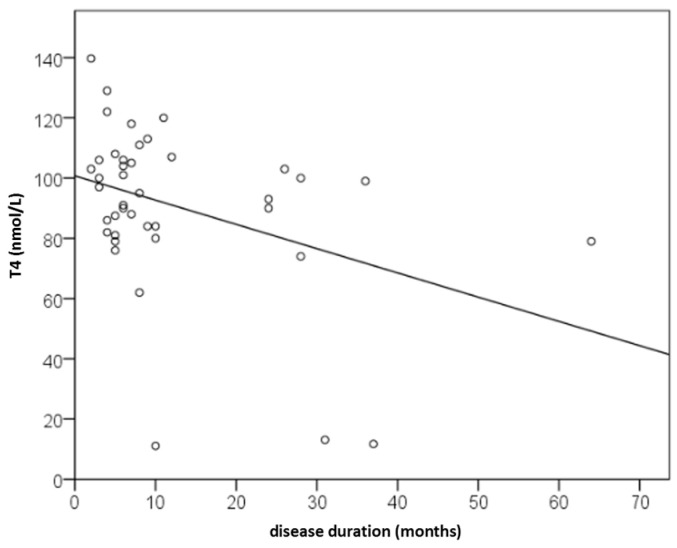
Dependence of serum thyroxine (T4, nmol/L) on CSU duration (months) (*p* = 0.036).

Among the examined serum factors, only thyroxine (T4) showed a significant dependence on disease duration, with a weak negative linear correlation (r = −0.328; *p* = 0.036; Figure 9). Thus, with increasing disease duration, T4 levels decreased. According to the regression equation y = 101 − 0.81x, each additional month of CSU reduced T4 levels by 0.81 nmol/L. T4 was significantly related to disease duration even when age and gender were controlled for (*p* = 0.024).

**Figure 10:**
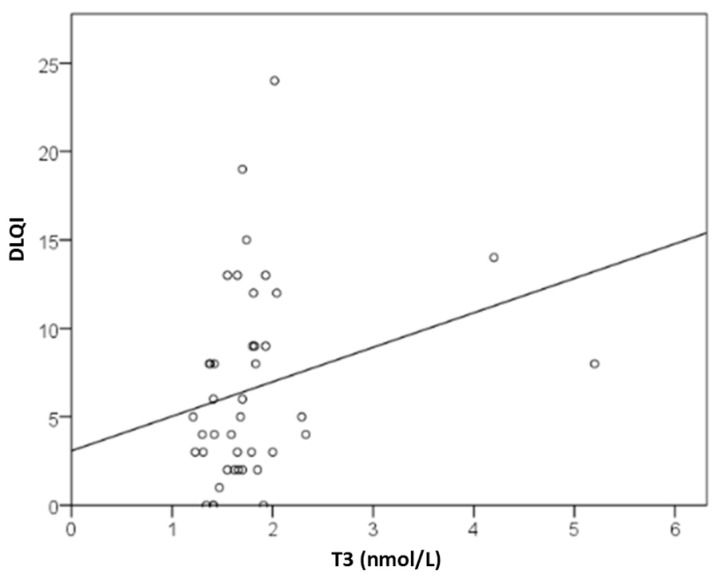
Association between serum T3 (nmol/L) concentration and impaired dermatological QoL measured by DLQI in CSU patients.

Values of T3 were moderately positively correlated with DLQI. Thus, each 1 nmol/L increase in T3 raised DLQI impairment by 1 point (y = 3.1 + 2.0x; r = 0.371; *p* = 0.017; Figure 10). However, no significant difference in T3 levels was observed when comparing dichotomized DLQI groups (median 1.6 vs. 1.7). In linear regression when controlling for the effects of age and gender and basophils, T3 lost its significance and was not related to DLQI. Notably, T4 was decreased in 27% of patients with moderate or severe CSU (daily UAS), but in none of the patients in remission or with mild disease (*p* = 0.010; V = 0.457). No significant linear association was found between daily UAS and T4 levels.

Changes in TSH levels were significantly and linearly correlated only with changes in CSU-specific QoL, as measured by the CU-Q2oL questionnaire (r = 0.655; *p* = 0.001). A greater reduction in TSH levels was associated with a greater improvement in QoL among patients with CSU.

After three months of standard antihistamine therapy, at T2, thyroid finding values did not differ significantly from baseline values (Table 3).

#### 2.3.4. Results for ANA

Autoimmune etiology of the disease (ANA titer ≥ 1:160) was confirmed in 22% of patients. An autoimmune background was present in only four patients. None of the patients in our study who exhibited elevated serum IL-6 levels had an autoimmune background (defined as ANA titer ≥ 1:160), whereas 17% of participants with normal serum IL-6 values did.

After three months of standard antihistamine therapy, the number of cases with ANA titer ≥ 1:160 did not change.

#### 2.3.5. Results for D-Dimer

Initially (at the T1 time point), serum D-dimer levels showed no significant correlation with disease duration, severity, or control of CSU, nor with patients’ QoL or other serum factors associated with CSU.

After three months of standard antihistamine therapy (at the T2 time point), serum D-dimer concentrations did not differ significantly from baseline values (Table 3).

#### 2.3.6. Results for Vitamin D

At the first measurement (T1), all participants had suboptimal serum vitamin D levels (<75 nmol/L), with a mean concentration of 61.0 nmol/L. Vitamin D deficiency was recorded in six patients (30%) with mild CSU and in six patients (28.6%) with moderate-to-severe CSU.

At the second measurement following a three-month course of vitamin D treatment, a significant increase in serum vitamin D concentration was recorded. In the patients who received vitamin D supplementation (due to recorded hypovitaminosis), their vitamin D levels increased substantially, accompanied by an improvement in clinical status, which was more frequently observed in this group compared with those who did not receive supplementation (69.8 vs. 58.1 nmol/L; *p* = 0.428, not statistically significant).

#### 2.3.7. Results for CRP

Initially, at T1, increased CRP was present in 8/41 (20%) of CSU patients.

After three months of CSU treatment, only CRP levels correlated with CSU control, as assessed by the UCT questionnaire (r = −0.459; *p* = 0.014). An increase in CRP values was associated with poorer CSU control (according to the UCT score).

#### 2.3.8. Results for ESR

For weekly disease severity measured by UAS7, none of the examined serum factors demonstrated statistical significance in receiver operating characteristic curve (ROC) analyses, but when they were dichotomized by reference values, ESR was the only significant factor. Although ESR did not show a linear association with CSU severity, it was less frequently reduced (14%) in patients with moderate/severe CSU (UAS7) than in those with remission or mild disease (45%) (*p* = 0.043). According to logistic regression, ESR was related to weekly UAS severity/UAS7 severity (OR 4.9; 95% CI 1.1–22.2; *p* = 0.038). Thus, ESR within the reference range was associated with a 4.9-fold higher likelihood of moderate or severe CSU compared to mild disease. None of the other examined serum factors were associated with UAS7 severity.

After three months of standard antihistamine therapy (at T2), ESR values did not differ significantly from baseline values (Table 3).

#### 2.3.9. Analysis of IL-6 Levels

Serum IL-6 showed a linear positive correlation with D-dimer (r = 0.606; *p* < 0.001), T3 (r = 0.370; *p* = 0.019), T4 (r = 0.321; *p* = 0.043), CRP (r = 0.379; *p* = 0.016) and ESR (r = 0.319; *p* = 0.045). IL-6 values did not linearly correlate with CSU severity (in any questionnaire) nor significantly differ between groups with different CSU severities, even when the groups were dichotomized. IL-6 values were mostly in the reference range (≤7 pg/mL); only four patients had elevated IL-6 values.

After three months of standard antihistamine therapy (at T2), IL-6 values did not differ significantly from baseline values (Table 3).

### 2.4. Results of Monitoring the Studied Factors and Molecules over Time (Longitudinal Data)

Analysis of the effect of standard antihistamine treatment on the values of the studied serum factors showed that during the patients’ monitoring and CSU treatment, there were no significant changes in the serum factors except in vitamin D values, as some patients were given supplements due to hypovitaminosis. Thus, after the 3-month treatment (at T2), the value of vitamin D had significantly increased, with a moderate effect size (*p* = 0.020; r = −0.446) (Table 3).

Observing clinical parameters during antihistamine treatment showed that this treatment significantly reduced CSU activity (daily UAS [*p* = 0.014; r = −0.464] and UAS7 [*p* = 0.015; r = −0.462] [moderate effect]) and increased the QoL of patients with CSU (i.e., reduced impairment) as measured by the DLQI questionnaire (*p* = 0.006; r = −0.519) (large effect size), but not the QoL specific to CU as measured by the CU-Q2oL questionnaire (Table 3).

CSU treatment also improved disease control as measured by the UCT questionnaire, with a large effect size (*p* = 0.005; r = −0.531).

Changes in basophil concentration were statistically significantly linearly correlated only with changes in daily CSU activity/daily UAS and impaired dermatological QoL (DLQI) (r = −0.470 and −0.385; *p* ≤ 0.043). A higher serum concentration of basophils was associated with a greater reduction in CSU activity and in impaired QoL in CSU patients.

Changes in TSH values were statistically significantly linearly correlated only with changes in CU-specific QoL (measured by the CU-Q2oL questionnaire) (r = 0.655; *p* = 0.001). Thus, a greater reduction in TSH values was associated with a greater reduction in impaired QoL in patients with CSU. Linear regressions showed that, when controlling for age and gender, the relationship between changes in basophil concentration, the UAS and DLQI was not significant. Change in TSH was significantly associated with CUQ2oL even when the effects of age and gender were controlled for in the regression (R = 0.675; R^2^ = 0.456; *p* = 0.010), and the independent contribution of TSH in explaining the variance was 3%.

After three months of CSU treatment, at the T2 time point, only the CRP value correlated with CSU control, as measured by the UCT questionnaire (r = −0.459; *p* = 0.014). In this case, with the increase in CRP values, CSU control decreased.

### 2.5. Key Statistically Significant Findings of This Research

Key statistically significant results obtained for serum factors significant in CSU patients’ condition are presented in Table 4. In addition, key findings recorded in CSU patients after 3 months of monitoring and antihistamine treatment (measured at the T2 time point) are presented in Table 5.

## 3. Discussion

While in our previous pilot study only three serum inflammatory molecules were assessed (IL-6, ESR and CRP) in a limited number of patients (20 patients), in this research we assessed other serum factors (including CBC, total IgE, thyroid antibodies and hormones, ANA, D-dimer, and vitamin D) in a greater number of CSU patients, with the aim to obtain more diagnostic data and temporal results during a longitudinal follow-up. Although several studies have examined potential biomarkers of CSU (including standard and additional molecules), prospective studies are very rare [31,32,33,34,35,36,37]. In this exploratory prospective study conducted on a small cohort, basophil concentration significantly negatively correlated with current CSU activity/daily UAS and impaired QoL (DLQI). Also, basopenia was more frequent in patients with current moderate/severe CSU than in those with mild disease or those who were in remission (measured by daily UAS), and basophil concentration was the only biomarker useful in assessing current CSU severity/daily UAS (sensitivity 78.6%; specificity 63%). According to the literature data, basophils are a clinically relevant indicator in CSU. In our study, basophil count was the only factor significantly associated with current CSU severity by daily UAS (sensitivity 78.6%, specificity 63%). Yanase et al. (2023) linked basophils with other CSU-related factors/biomarkers (e.g., plasma histamine, total IgE) that are useful for defining disease activity [38]. De Montjoye et al. (2021) likewise reported a positive correlation between UAS7 and CRP and a negative correlation between CSU activity and basophil count [36]. In pooled analyses of omalizumab trials, Poddighe et al. reported increases in circulating basophils during conventional-dose treatment [39,40,41,42]. Regarding omalizumab response prediction, Cakmak et al. (2022) found that blood eosinophil and basophil counts and total IgE were significant predictors of treatment response in CU [43]. In general, in CSU, total IgE is primarily an indicator of response to therapy and is rarely associated with the condition of CSU, as noted in our study in which its values did not change during monitoring. In general, according to most data from the literature, blood basopenia is a significant indicator of increased CSU activity/severity due to its accumulation in skin lesions. High levels of serum IgE in CSU indicate a better response to omalizumab, while total IgE values are associated with autoimmune disease (type IIb) and a poor response to omalizumab.

Furthermore, in CSU patients, according to most data from the literature, elevated D-dimer, a key marker of coagulation/fibrinolysis activation, often indicates high CSU severity, antihistamine resistance, and summer exacerbation. De Montjoye et al. showed D-dimer and CRP to be positively correlated in CSU, and that in autoimmune CSU, D-dimer was higher and basophils lower, and that during omalizumab therapy D-dimer and CRP were significantly decreased [36]. In our study, D-dimer did not correlate with CSU duration, severity, control, QoL or with other examined CSU-related factors [42,44,45]. Additional analysis of CSU-related serum factors also warrants consideration. According to our results, only T4 significantly depended on CSU duration. One meta-analysis by Zhang et al. (2022) found that CSU patients had markedly higher odds of positive thyroid findings than HCs [44]. Regarding the activity and severity of CSU, during the active phase of CSU higher serum values of D-dimer, CRP and IL-6 were confirmed, while during CSU remission they were lower. Also, high activity of CSU (UAS7) correlates with elevated D-dimer, CRP and prothrombin fragment 1+2 (F1+2). High levels of D-dimer and CRP are often markers of resistance to standard antihistamine therapy.

Concerning the clinical value of assessment of ANA in CSU patients, Arunkajohnsak et al. (2022) found that in ANA-positive CSU, there was higher ESR and a lower prevalence of allergic conditions such as rhinitis [46]. In our study, most patients had no autoimmune background (78%); 22% had an autoimmune background (ANA ≥ 1:160), which was not associated with CSU severity. The literature indicates that ANA is more often positive in CSU, but overt autoimmune diseases may be diagnosed up to 10 years after CSU onset, which may explain the absence of a significant association between ANA titer and CSU in our sample [18]. Also, an association between CSU, autoimmunity and gender should be mentioned. The number of female subjects in our study (32/41) confirms the epidemiological findings that women are more often affected by CSU, a possible reason for this lying in the chronic nature and inflammatory genesis of the disease, as well as a possible autoimmune basis. However, values of ANA were stable/unchanged in our patients, supporting previous study results.

Most previous research results have shown lower vitamin D values in CSU than in HCs, and our research results support reduced vitamin D values [47,48,49,50,51]. According to one research work which examined vitamin D concentrations across multiple dermatoses (CSU, atopic dermatitis, contact dermatitis), vitamin D tended to be lower in CU (not significantly) and did not differ significantly by age, sex, residence, or allergy presence/type [52]. In general, according to most of the literature data, low serum vitamin D levels are frequently observed in patients with CSU and correlate with greater severity of CSU and longer CSU duration.

Concerning inflammatory molecules valuable in CSU diagnostics, for monitoring active CU inflammation generally, CRP is considered a better and more sensitive indicator than ESR. Its advantages for CSU diagnostics are better sensitivity, faster response (CRP levels rise and fall more rapidly than ESR, making it better for tracking treatment response), specificity (CRP is a direct measure of an inflammatory liver-derived protein in response to IL-6, while ESR is an indirect measure influenced by plasma viscosity, erythrocyte shapes, and other non-inflammatory factors), and predictive value (higher CRP levels are linked to higher CSU activity, poorer QoL, and reduced response to H1-antihistamines). However, ESR may better correlate with indicators of CSU severity; e.g., in our pilot study, ESR was a good indicator of weekly CSU severity/UAS7, which was confirmed in this expanded study. Concerning other previous studies on inflammatory molecules in CSU (from different countries), for instance, Kasperska-Zajac et al. (2013) found higher CRP in CSU subjects than in controls and a positive correlation between CRP and procalcitonin (absent in controls) [35]. In one prospective study, de Montjoye et al. (2021) observed that weekly CSU activity (UAS7) correlated positively with CRP and negatively with peripheral blood basophils [36]. Kolkhir et al. reported higher CRP in antihistamine-nonresponders than responders and correlations between CRP and other inflammatory markers (ESR, leukocytes/neutrophils, IL-6) [37]. Ucmak et al. (2013) found associations between UAS and IL-6/CRP, but no correlation between IL-6 and CRP [11]. In our CSU patients, serum IL-6 values significantly correlated with other serum factors (D-dimer, T3 and T4, CRP, ESR), where these examined serum factors may reflect CSU activity and control, as well as QoL impairment, as assessed by specific questionnaires [30,31,32,33,34]. Although in our pilot study IL-6 correlated with the once-daily UAS and DLQI, this expanded study did not confirm this. According to the literature data, significantly higher CRP levels were confirmed for CSU patients than in HCs (which is, biologically, expected), and there was a significant correlation between IL-6 and CRP [10]. Our findings align with these results, showing a significant positive linear correlation between IL-6 and CRP, and also support our pilot study results with similar findings [30]. Another study on CSU also showed higher IL-6 in CSU patients, while CRP did not differ significantly from controls [34]. In another study, IL-6 was significantly higher in CSU than in HCs; IL-6 correlated with weekly CSU activity/UAS7, while in mild CSU IL-6 did not differ from HCs, and these findings support IL-6 as a potential indicator of CSU activity [10]. In our research, high IL-6 values were recorded in moderate/severe CSU more frequently than in CSU remission or mild CSU. This is consistent with data that IL-6 is an early pro-inflammatory cytokine in systemic inflammation, with higher levels observed in more severe CSU (measured by daily UAS at the time of serum sampling). The lack of statistical significance likely reflects the small number of severe cases and ongoing antihistamine use.

Finally, the strengths of our study are its prospective character—prospective studies on CSU are very rare—and the obtained data on meaningful use of clinical indices during follow-up for insight into CSU clinical conditions (UAS7, daily UAS, UCT, DLQI). Also relevant is the data that basophil concentration was the only examined serum factor useful in assessing current CSU severity/daily UAS (sensitivity 78.6%; specificity 63%) and that basopenia was more frequent in patients with current moderate/severe CSU than in those with mild disease or those who were in remission (daily UAS) (79% vs. 37%). Furthermore, T4 values showed a significant dependence on CSU duration, and ESR was the only examined serum factor significantly associated with weekly CSU severity (UAS7). However, the following limitations of this research should be emphasized: the low number of participants and predominance of mild CSU (which probably affected study results), and the data that some of our patients had recently been treated with systemic corticosteroids or had used antihistamines concurrently. Also, the dropout rate at the second follow-up limits generalizability; the 32% dropout could affect both the statistical power and the validity of the results, and could have introduced dropout bias. Also, given the multiple correlation analyses performed, false positive findings are possible. In addition, measured serum factors are influenced by several factors such as pathogenetic mechanisms, environmental conditions and patient characteristics. Thus, some patients’ characteristics may have changed their individual results, and thus may be important. Aside from age and gender, for which we controlled in our study, features such as body mass index and obesity (which is associated with lower total IgE levels and poor response to omalizumab and increased systemic inflammation) can affect results. Also, there are different immune CSU endotypes, including type IIb (autoimmune CSU), in which patients with IgG autoantibodies often show specific profiles (including low total IgE and basopenia), and type I (autoallergic CSU), often associated with normal to high IgE values (and a better response to omalizumab) and autoantibodies; anti-TPO is associated with more severe, long-lasting, and autoimmune CSU. In addition, CU condition could be influenced by seasonal/environmental variations e.g., during summer months, higher CU activity could result in changes in serum factors (elevated D-dimer, CRP, and leukocytes and in a higher neutrophil to lymphocyte ratio); and there are also stronger effects of air pollutants, especially exposure to ozone, high levels of which are associated with poorer CSU control, affecting inflammatory molecules [53]. Finally, coagulation factors may be important for disease activity. Thus, there are various factors that are difficult to measure that could have affected our results. For this reason, our study needs to be supported by larger studies.

## 4. Materials and Methods

### 4.1. Subjects

Our prospective study was conducted at the Department of Dermatovenereology, University Hospital Center “Sestre Milosrdnice,” Zagreb, Croatia, from November 2021 to March 2023. Inclusion criteria were age ≥ 18 years and a confirmed diagnosis of CSU with daily or near-daily wheals or angioedema for at least 6 weeks. Exclusion criteria included acute urticaria, urticarial vasculitis, other non-CU subtypes, inducible urticaria unrelated to CSU, isolated angioedema, systemic diseases affecting assessment, and use of immunosuppressive drugs, psychoactive therapy, biologics, or systemic corticosteroids within two weeks prior to enrollment.

A total of 41 adults with CSU were enrolled (32 women, 9 men; age range 23–79 years). In 44% of participants, the disease had been present for 3–6 months. All patients were treated with bilastine (20 mg twice daily, increased up to four tablets daily during exacerbations).

### 4.2. The Ethical Statement

Our research was approved by the Ethics Committee of the University Hospital Center “Sestre Milosrdnice”, Zagreb, Croatia, in November 2021 (number of protocol: 251-29-11-21-01-9), and all patients included in the study gave their written informed consent to participate.

### 4.3. Methods

Blood samples were collected to measure serum CBC, total IgE, thyroid findings, ANA, D-dimer, and vitamin D, as well as CRP, ESR, and IL-6. Participants also completed standardized assessment tools for CSU severity: the daily UAS (day of sampling), UAS7 (previous 7 days), UCT (previous 4 weeks), and DLQI (previous 7 days).

#### 4.3.1. Serum Parameters

From a whole blood sample collected by venipuncture into an ethylenediaminetetraacetic acid (EDTA) tube, a CBC was performed, specifically determining the concentration and proportion of basophils in the blood. Basophil values were expressed both as an absolute count and as a percentage of the total leukocyte count. Basopenia was defined as a basophil count lower than 0.1 × 10^3^/mm^3^.

For the determination of total IgE, a blood sample was collected into additive-free tubes (Vaccuette^®^, Greiner Bio-One, Kremsmünster, Austria) and centrifuged, and total IgE levels were measured from serum using a chemiluminescent assay with the Immulite 2000 analyzer (Siemens Healthcare Diagnostics Products GmbH, Marburg, Germany). Reference values for total IgE were ≤114 kIU/L.

For the determination of thyroid findings, a blood sample was collected into additive-free tubes and centrifuged, and serum concentrations of the aforementioned parameters were measured using a Cobas e601 immunoassay analyzer (Roche Diagnostics, Basel, Switzerland).

For the determination of ANA titers, a blood sample was collected into additive-free tubes (Vaccuette^®^, Greiner Bio-One, Kremsmünster, Austria) and centrifuged, and ANA determination was performed using the indirect immunofluorescence method on HEp-2 cells, which are considered the gold standard for ANA detection. The antihuman immunoglobulin conjugate labeled with the fluorochrome fluorescein isothiocyanate (FITC) used in the ANA IIF test is specific for IgG-class antibodies. For interpretation, ANA titers below 1:160 were considered negative, whereas patients with CSU who had an ANA titer ≥1:160 were considered to have an underlying autoimmune disorder as one of the possible etiopathogenetic causes of CSU.

For the determination of D-dimer, a blood sample was collected into a sodium citrate tube (Vaccuette^®^, Greiner Bio-One, Kremsmünster, Austria), and values were determined from plasma.

For the determination of vitamin D from serum, a blood sample was collected into an additive-free tube (Vaccuette^®^, Greiner Bio-One, Kremsmünster, Austria) and centrifuged, and the serum vitamin D concentration was measured using the commercially available Elecsys^®^ Vitamin D assay on a Cobas e601 immunoassay analyzer (Roche Diagnostics, Basel, Switzerland). Some patients, for whom low vitamin D values were recorded, were supplemented according to each patient’s specific deficit.

CRP, ESR, IL-6 and other serum markers were analyzed using standard laboratory methods. ESR was determined by the Westergren technique using citrate-treated blood in Vaccuette^®^ tubes. CRP was measured on the Architect c8000 analyzer (latex immunoturbidimetry; reference range 0–5 mg/L). Serum IL-6 was quantified by electrochemiluminescence with the Roche Cobas E601 system (reference range 0–7 pg/mL).

#### 4.3.2. Questionnaires on Clinical CSU Features

To assess CSU severity and patient QoL, we used the UAS7, once-daily UAS, UCT, DLQI, and CU-Q2oL, in accordance with current guidelines [1].

The Urticaria Activity Score (UAS) evaluates wheals and pruritus over a 24 h period [1,30].

The UCT consists of four questions evaluating symptom burden, QoL impact, medication effectiveness, and overall disease control over the preceding four weeks, with a total score of 0–16 (≥12 indicates well-controlled urticaria, whereas <12 reflects poor control) [31].

The DLQI covers six domains (symptoms, daily activities, leisure, work/school, relationships, and treatment) over a 7-day recall [32].

CU-Q2oL is specifically designed to assess QoL impairment in CSU patients. It consists of 23 questions across six domains [1,33].

#### 4.3.3. Statistical Analysis

Data normality was assessed using the Kolmogorov–Smirnov and Shapiro–Wilk tests. Associations were analyzed with Spearman’s rank correlation, and Cohen’s criteria were used for interpretation: r = 0.25–0.3 weak, 0.3–0.5 moderate, 0.5–0.7 strong, >0.7 very strong. For the comparison of correlation coefficients, r-to-z transformations and the z-test were used.

Examined serum factors were compared across disease categories using the Kruskal–Wallis and Mann–Whitney tests.

Univariate and multivariate logistic and linear regression analyses were employed to evaluate the relationship between the examined serum factors and the severity of CSU and QoL when confounders were controlled for. The sensitivity and specificity of each examined serum factor significantly associated with clinical severity were assessed using ROC analysis, which was done only for the hypothetical examined serum factors for the daily UAS, basophils, CRP, IL-6, and D-dimers, which are more indicative of the current state for that day or several days, and for the weekly UAS7 in ESR.

Comparison of initial (T1) and follow-up (T2) findings was conducted using the Wilcoxon test, with effect size quantified as for the Mann–Whitney test. Imputation of missing data was not performed. Instead, a complete-case analysis was conducted, including only those cases with complete data for the variables relevant to each specific analysis. Baseline characteristics did not differ significantly between completers and non-completers.

All analyses were performed using IBM SPSS Statistics software, version 22.0 (IBM Corp., Armonk, NY, USA), with the level of statistical significance set at *p* < 0.05.

A minimum sample size of 28 subjects was calculated with an expected difference in IL-6 due to therapy of five with a standard deviation of nine with a power of 80% and a significance level of 0.05. Due to an expected dropout rate of 20%, the sample was increased accordingly to 35 subjects. The following online calculator was used for this calculation: https://statulator.com/SampleSize/ss2PM.html, accessed on 15 October 2021.

## 5. Conclusions

Since the results of this primarily exploratory and hypothesis-generating study with CSU patients confirmed a decreased basophil count, especially in moderate/severe CSU, this supports basophil assessment as useful in clinical practice for insight into disease status. Basophil concentration negatively correlated with patients’ current CSU condition and with QoL/DLQI, and was the only factor useful in assessing current CSU severity (daily UAS). Also, ESR was the only significant factor related to the weekly CSU severity/UAS7, while T4 showed a significant dependence on CSU duration. We support diagnostics using basophil counts and clinical indices (UAS7, daily UAS, UCT, DLQI) for insight into patients’ clinical CSU condition during their follow-up. Other factors’ values predominantly did not change over the 3-month treatment period, so it is not useful to measure them repeatedly. Given the limitations of our study that should be taken into account, our results support the use of a single daily UAS to determine the current severity of CSU, basophil count to assess the severity of CSU (daily UAS), and serum IL-6 assessment in routine diagnostics of CSU patients. Although this exploratory study involved a small cohort and has many limitations, these promising results highlight the need for replication in a study with a greater number of CSU patients.

## Figures and Tables

**Table 1:** Descriptive statistics of patients with CSU (initial sample at T1).

	Variable	N	Mean ± SD	Median	IQR	Min	Max
Clinical features	Age	41	42.9 ± 16.2	39	30–55	21	79
	Disease duration (months)	41	12.2 ± 12.8	7	5–11.5	2	64
	Daily UAS	41	2.1 ± 1.9	2	0–3.5	0	6
	UAS7	41	15.7 ± 10.0	16	7.5–23.5	0	39
	DLQI	41	6.6 ± 5.5	5	2.5–9	0	24
	CU-Q2Ol	35	29.9 ± 20.3	23	15–47	0	85
	UCT	41	8.2 ± 2.6	9	6–10	3	14
Serum factors	Percentage of basophils (%)	41	0.66 ± 0.36	0.50	0.35–0.80	0.0	1.7
	Basophil concentration (×10^9^/L)	41	0.03 ± 0.4	0.00	0.00–0.09	0.00	0.10
	Anti-TG (kIU/L)	41	24.3 ± 107.3	0.9	0.7–2.3	0.3	677.3
	D-dimer (mg/L)	39	0.48 ± 0.48	0.32	0.21–0.54	0.10	2.73
	Total IgE (kIU/L)	41	203.1 ± 396.7	94.0	18.5–171.0	3.0	2292.0
	Vitamin D (nmol/L)	41	60.3 ± 24.3	61.0	43.5–72.5	23.0	113.0
	ESR (mm/3.6 ks)	41	8.9 ± 6.9	6.0	4.0–12.5	2.0	28.0
	Serum IL-6 (pg/mL)	40	3.6 ± 2.9	2.6	1.8–4.4	1.5	12.8
	CRP (mg/L)	41	3.0 ± 3.5	1.5	0.7–3.7	0.2	14.0

Min—minimum value; Max—maximum value.

**Table 2:** Relationships between examined serum factors and CSU duration, severity, control, and QoL recorded at the initial T1 time point.

Variables		Age (y)	Disease Duration (mo.)	Daily UAS	UAS7	DLQI	CU-Q2oL	UCT
Percentage of basophils (%)	r	0.082	0.18	−0.205	−0.284	−0.282	**−0.385**	0.104
	*p*	0.610	0.26	0.198	0.072	0.074	**0.023**	0.518
	N	41	41	41	41	41	35	41
Basophil concentration (× 10^9^/L)	r	−0.049	−0.018	**−0.475**	−0.277	**−0.358**	**−0.359**	−0.015
	*p*	0.763	0.913	**0.002**	0.08	**0.021**	**0.034**	0.926
	N	41	41	41	41	41	35	41
D-dimers (mg/L)	r	0.194	−0.004	0.116	0.193	0.128	0.16	0.079
	*p*	0.236	0.982	0.48	0.238	0.439	0.374	0.63
	N	39	39	39	39	39	33	39
Total IgE (kIU/L)	r	−0.125	0.139	0.049	0.144	−0.019	−0.209	0.064
	*p*	0.436	0.384	0.759	0.369	0.907	0.228	0.692
	N	41	41	41	41	41	35	41
TSH (mIU/L)	r	−0.046	−0.184	−0.048	−0.144	0.079	0.259	0.158
	*p*	0.780	0.255	0.77	0.375	0.628	0.139	0.329
	N	40	40	40	40	40	34	40
T3 (nmol/L)	r	−0.154	−0.166	0.11	0.266	**0.371**	0.326	−0.28
	*p*	0.338	0.299	0.495	0.093	**0.017**	0.056	0.076
	N	41	41	41	41	41	35	41
T4 (nmol/L)	r	−0.050	**−0.328**	−0.229	0.016	0.068	−0.079	0.056
	*p*	0.754	**0.036**	0.149	0.92	0.672	0.652	0.727
	N	41	41	41	41	41	35	41
Anti-TPO (kIU/L)	r	0.092	0.031	−0.034	0.017	0.055	0.102	−0.067
	*p*	0.572	0.849	0.834	0.916	0.735	0.565	0.68
	N	40	40	40	40	40	34	40
Anti-TG (kIU/L)	r	−0.055	0.234	0.058	0.001	0.007	0.087	0.011
	*p*	0.732	0.141	0.721	0.997	0.965	0.619	0.945
	N	41	41	41	41	41	35	41
Vitamin D (nmol/L)	r	**0.526**	0.178	0.172	−0.024	−0.041	0.03	0.23
	*p*	**<0.001**	0.265	0.282	0.883	0.798	0.865	0.147
	N	41	41	41	41	41	35	41
IL−6 (pg/mL)	r	0.305	−0.037	0.114	0.087	0.135	0.160	−0.068
	*p*	0.056	0.820	0.483	0.564	0.405	0.366	0.678
	N	40	40	40	40	40	40	40
CRP (mg/L)	r	0.171	−0.022	−0.06	0.195	0.303	0.309	−0.198
	*p*	0.286	0.894	0.709	0.221	0.054	0.071	0.215
	N	41	41	41	41	41	35	41
ESR (mm/3.6 ks)	r	**0.392**	0.278	0.013	0.181	0.152	0.267	−0.011
	*p*	**0.011**	0.078	0.935	0.257	0.343	0.122	0.944
	N	41	41	41	41	41	35	41

*p*—level of statistical significance, r—Spearman’s correlation coefficient, N—number of patients with CSU, mo.—months, daily UAS—daily urticaria activity questionnaire, UAS7—urticaria activity questionnaire during 7 consecutive days, DLQI—dermatological quality of life index, CU-Q2oL—quality of life questionnaire specific for chronic urticaria, UCT—urticaria control test, CRP—C-reactive protein, ESR—erythrocyte sedimentation rate, IgE—immunoglobulin E, TSH—thyroid-stimulating hormone, T3—triiodothyronine, T4—thyroxine, anti-TPO—thyroid peroxidase antibodies, anti-TG—thyroglobulin antibodies.

**Table 3:** Comparison of the examined parameters before and after treatment (at the T1 and T2 time points).

		N	T1 Median (IQR)	T2 Median (IQR)	*p* *	r **
Clinical features	Daily UAS	28	1.5 (0–4)	0.5 (0–2)	**0.014**	−0.464
	UAS7	28	11 (6.3–22)	7 (1–15)	**0.020**	−0.500
	DLQI	28	4 (2–8)	2 (1–6)	**0.006**	−0.529
	CU-Q2oL	22	21 (13.5–36)	14.5 (8–26)	0.068	−0.389
	UCT	28	9 (6.3–9.8)	9.5 (8–12)	**0.005**	0.841
Serum factors	Vitamin D (nmol/L)	27	59.0 (44.0–74.0)	75.0 (53.0–98.0)	**0.020**	−0.446
	Basophils (%)	28	0.55 (0.33–0.90)	0.50 (0.33–0.78)	0.378	−0.167
	Basophils (×10^9^/L)	28	0.02 (0.00–0.10)	0.03 (0.02–0.05)	0.889	−0.027
	D-dimers (mg/L)	27	0.32 (0.21–0.58)	0.34 (0.23–0.51)	0.777	−0.055
	Total IgE (kIU/L)	27	54.0 (18.0–149.0)	69 (24.0–161.0)	0.501	−0.130
	TSH (mIU/L)	27	1.8 (1.1–3.0)	2.2 (1.2–3.1)	0.501	−0.130
	T3 (nmol/L)	27	1.6 (1.4–1.8)	1.5 (1.4–1.8)	0.819	−0.044
	T4 (nmol/L)	27	90.0 (81.0–106.0)	94.0 (84.0–103.0)	0.486	−0.134
	Anti-TPO q (kIU/L)	28	1.8 (0.7–4.5)	1.7 (0.6–4.6)	0.269	−0.209
	Anti-TG (kIU/L)	27	0.9 (0.7–1.8)	1.0 (0.6–2.4)	0.850	−0.036
	Serum IL-6 (pg/mL)	26	2.5 (1.9–4.6)	3.4 (1.5–5.9)	0.841	−0.039
	CRP (mg/L)	28	1.4 (0.5–3.4)	1.4 (0.6–2.9)	0	0
	ESR (mm/3.6 ks)	28	6.0 (4.0–9.5)	8.0 (4.0–12.0)	0.434	−0.148

* Wilcoxon test; ** effect size; IQR—interquartile range.

**Table 4:** Key statistically significant results obtained for serum factors significant in CSU patients’ condition (initial serum findings measured at the T1 time point).

Factors	Significant Results
Basophils	Basopenia was more common in moderate/severe CSU than in mild CSU/remission (measured by daily UAS) (79% vs. 37%) (*p* = 0.020) and was associated with a 6.2-fold increased likelihood of moderate/severe CSU (*p* = 0.017).
	Basophil level was the only examined factor associated with current CSU severity/daily UAS (78.6% sensitivity, 63% specificity). Basophil levels had a significantly negative linear correlation with daily CSU activity/daily UAS (*p* = 0.002).
	Basophil counts significantly correlated with impaired QoL (DLQI) (*p* ≤ 0.034).
	There was a stronger association between basophil concentration/levels and QoL impairment/CU-Q2oL than between basophil proportion and QoL impairment/CU-Q2oL (*p* ≤ 0.023).
Thyroid findings	Among the analyzed factors, only thyroxine (T4) significantly depended on CSU duration (a weak negative linear correlation; *p* = 0.036).
	Changes in TSH levels significantly and linearly correlated only with changes in QoL/the CU-Q2oL (*p* = 0.001).
Inflammatory molecules ESR, IL-6, CRP	ESR was the only significant factor related to weekly CSU severity/UAS7 (*p* = 0.038) (logistic regression). Serum IL-6 showed a linear positive correlation with CRP, ESR, D-dimer, T3 and T4.

**Table 5:** Key significant findings recorded in CSU patients after 3 months of monitoring and antihistamine treatment (recorded at the T2 time point).

	Factors	Significant Results
Clinical features (indices)	UAS	Antihistamine treatment significantly reduced current and weekly CSU activity, as measured by daily UAS and weekly UAS/ UAS7 (*p* = 0.014; *p* = 0.015) (moderate effect).
	QoL	Antihistamine treatment increased the QoL of patients (measured by DLQI) (*p* = 0.006) (large effect size).
	UCT	Antihistamine treatment significantly improved CSU control (UCT) (large effect size) (*p* = 0.005).
Serum factors	Basophils	Changes in basophil concentration significantly linearly correlated only with changes in daily CSU activity/daily UAS and impaired dermatological QoL/DLQI (*p* ≤ 0.043). A higher serum basophil concentration was associated with a greater reduction in both CSU activity and impaired QoL in CSU patients.
	Thyroid findings	Changes in TSH values significantly linearly correlated only with changes in CU-specific QoL (measured by the CU-Q2oL) (r = 0.655; *p* = 0.001). Change in TSH was significantly associated with CU-Q2oL (*p* = 0.010).
	Vitamin D	After antihistamine treatment, the value of vitamin D significantly increased, with a moderate effect size (*p* = 0.020; r = −0.446). (Some patients with recorded hypovitaminosis D were supplemented.)
	CRP	After antihistamine treatment, only the CRP value correlated with CSU control (UCT, *p* = 0.014).

## Data Availability

The original contributions presented in this study are included in the article. Further inquiries can be directed to the corresponding authors.
